# Advances in olfactory augmented virtual reality towards future metaverse applications

**DOI:** 10.1038/s41467-024-50261-9

**Published:** 2024-07-31

**Authors:** Zixuan Zhang, Xinge Guo, Chengkuo Lee

**Affiliations:** 1 Department of Electrical and Computer Engineering, National University of Singapore, 4 Engineering Drive 3, Singapore, 117576 Singapore; 2 Center for Intelligent Sensors and MEMS (CISM), National University of Singapore, 4 Engineering Drive 3, Singapore, 117576 Singapore; 3 NUS Graduate School - Integrative Sciences and Engineering Programme (ISEP), National University of Singapore, 21 Lower Kent Ridge Road, Singapore, 119077 Singapore

**Keywords:** Electrical and electronic engineering, Biomedical engineering

## Abstract

Recent advances in virtual reality technologies accelerate the immersive interaction between human and augmented 3D virtual worlds. Here, the authors discuss olfactory feedback technologies that facilitate interaction with real and virtual objects and the evolution of wearable devices for immersive VR/AR applications.

Virtual reality (VR) technology typically employs visual and auditory devices, such as head-mounted displays and VR goggles, but further immersion in virtual spaces can be achieved through wearable devices including gloves, exoskeletons, shoes, etc., and electronic skins (e-skin)^1^. These wearable devices enable the realization of full-body sensory perception or sensation, requiring the capability to perceive human movements, simulate sensations, and being flexible and comfortable for users (Fig. 1). Recently, sensors for detecting physical signals, e.g., stretch, pressure, temperature, etc., have been flourishing. They can be utilized for biosensing and detecting human motion signals^2^. In addition, wearable systems integrating multiple types of flexible actuators, such as tendon actuators, pneumatic actuators, and electrostatic actuators, can achieve kinesthetic, electro-tactile, vibrotactile, and thermal-tactile feedback, respectively. Such systems provide tactile stimulation to the skin’s sensory receptors^3^. Multimodal haptic feedback enables users a better immersive sensory experience by enhancing interaction between humans and virtual environments. For example, an augmented tactile-perception and haptic feedback ring, equipped with multimodal sensing encompassing tactile, temperature sensing, and feedback encompassing vibratory and thermal haptic stimuli, demonstrates the potential beyond the commercial rings^4^. A skin-integrated wireless haptic interface using actuator arrays offers multimodal mechanisms for complex feedback. Within this interface, various feedback modes including mechanical, electrotactile, and thermal, are utilized to target distinct cutaneous receptors selectively, offering users diverse haptic sensations^5^. The fusion of VR display technology with such multimodal somatosensory sensation for full-body perception and feedback is essential for future metaverse applications. An augmented 3D virtual society, i.e., a 4D VR world, could facilitate intelligent and interactive experiences across various domains such as social interactions, education, entertainment, healthcare, etc.

**Fig. 1:**
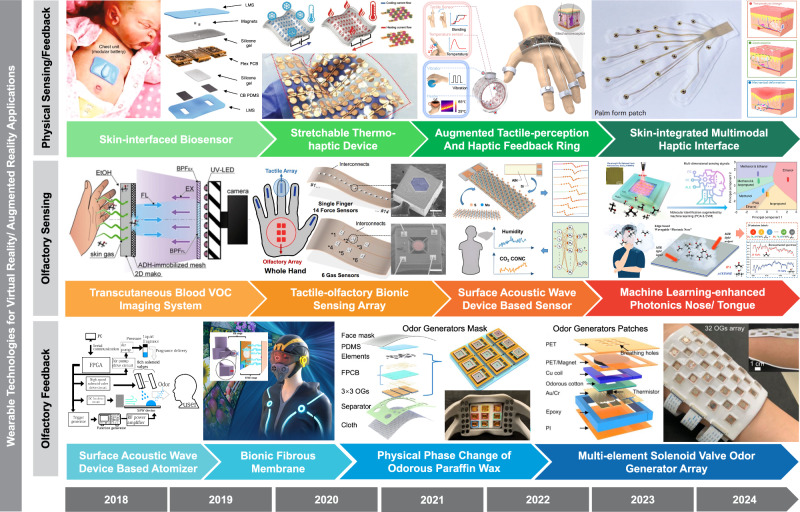
Evolution of wearable technologies for enhancing the immersive interaction in virtual reality/ augmented reality applications. The development trends of physical sensing and physical feedback devices^2–5^, olfactory sensing devices^6–10^, and olfactory feedback devices^11–14^. Figure adapted with permission from: (‘Skin-interfaced biosensor’), ref. ^2^, Springer Nature Ltd; (‘Stretchable thermos-haptic device’), ref. ^3^, Wiley; (‘Augmented tactile-perception and haptic feedback ring’), ref. ^4^, Springer Nature Ltd; (‘Skin-interfaced multimodal haptic interface’), ref. ^5^, Springer Nature Ltd; (‘Transcutaneous blood VOC imaging system’), ref. ^6^, American Chemical Society; (‘Tactile-olfactory bionic sensing array’), ref. ^7^, Springer Nature Ltd; (‘Surface acoustic wave device based sensor’), ref. ^8^, American Chemical Society; (‘Machine learning-enhanced mid-infrared gas sensing’, top), ref. ^9^, Springer Nature Ltd; (‘Machine learning-enhanced mid-infrared gas sensing’, bottom), ref. ^10^, Wiley; (‘Surface acoustic wave device based atomizer’), ref. ^11^, IEEE; (‘Bionic fibrous membrane’), ref., Wiley; (‘Physical phase change of odorous paraffin wax’), ref. ^13^, Springer Nature Ltd; (‘Multi-element solenoid value array’), ref. ^14^, Springer Nature Ltd.

Equally important to traditional visual, auditory, and tactile sensations, olfaction exerts both physiological and psychological influences on humans. The pivotal role of olfaction in shaping human experiences is undeniable, given that many aspects of daily life that are influenced by scent emanate from industrial processes, transportation, household products, etc. With significant advancements in chemistry, biology, and neuroscience, odor sensors (gas/ liquid sensors) are witnessing the rapid development of decoding complex odor mixtures facilitated by conventional rigid and innovative flexible sensing electronics. They provide non-invasive methods for detecting biomarkers and informing about metabolic processes and disease progression for humans and plants, thus greatly appealing for real-time health monitoring and point-of-care diagnostics. A highly sensitive bio-fluorometric gas sensor “bio-sniffer” based on an enzymatic reaction is developed to measure the concentration of transcutaneous ethanol (EtOH) after drinking^6^. Concurrently, the emergence of artificial intelligence (AI)-based data analytics introduces the potential to enhance sensor functionalities towards intelligent sensing. Leveraging the robust feature extraction capabilities of machine learning, previously subtle valuable features within complex signal outputs can be discerned and harnessed, which can be used to achieve advanced sensory perceptions. A tactile-olfactory sensing array is developed to permit the real-time acquisition of the local topography, stiffness, and odor of various objects without visual input^7^, by leveraging the bioinspired olfactory-tactile associated AI algorithm. Moreover, a single piezoelectric cantilever is used to detect temperature and CO_2_ concentration with the interference of humidity and temperature for noncontact sensation and monitoring human breath and plant ecosystem^8^. Photonics noses/tongues are the emerging vital tools that utilize optical technology to mimic human olfactory systems. The mid-infrared photonics nose/ tongue distinguishes different olfactory by analyzing the absorption spectra of liquid gas samples^9,10^. They offer advantages such as high sensitivity, rapid response, and noncontact detection, making them valuable in medical, food industry, and environmental monitoring applications. The development and application of artificial noses and tongues with better-than-human capability in AI-enhanced optical sensing technology are crucial for enhancing the sensitivity and accuracy of odor and taste detection.

Unlike the visual, auditory, and tactile sensory channels, olfaction is a nonlinear chemical sense, which makes it challenging to develop a technically comprehensive olfactory feedback system for precise control of odor generation and delivery. The olfactory feedback technologies reported so far still face huge challenges. Current olfaction-generating technologies are struggled with bulky dimensions, limited scent variety, and slow response times. Such olfaction-generating technologies are mainly associated with either large instruments designed to generate smell in a closed area/ room or an in-built bulky VR set. Consequently, these olfaction feedback approaches are far behind the advancement of visual/ auditory-based VR devices, thereby severely constraining their potential applications. For example, an olfactory display for blending many ingredients in any recipe was developed based on solenoid valves and surface acoustic wave atomizers^11^. However, the bulky circuits and systems make it difficult for them to become wearable solutions. In addition, a bionic fibrous membrane (BFM) integrated with the function of electrostatic field accelerated evaporation is applied to realize the virtual olfactory generation system^12^. Although it realizes the functionality of wearable and 4-odor generation, the response time of this system still needs to be improved. Therefore, Liu et al. reported a skin-interfaced olfactory feedback system with wirelessly programmable capabilities based on an array of flexible and miniaturized odor generators (OGs) for olfactory VR applications^13^, which demonstrates outstanding performance including rapid response rates to odor concentration, prolonged continuous operation, robust mechanical/ electrical stability, and minimal power consumption. Furthermore, they developed an OGs array with advanced artificial intelligence (AI) algorithms^14^, which exhibit milestone advances in various features of performance, including millisecond-level response time (70 ms), milliwatt-scale power consumption (84.8 mW), miniaturized size (11 mm × 10 mm × 1.8 mm), and high stability (12-hr continuous operation). Miniaturized olfactory generators with millisecond-level response times, milliwatt-scale power consumption, compact size, stability, and a high number of odor supplies establish a bridge between electronics and users for broad applications ranging from entertainment to education, medical treatment, and human-machine interfaces. Compared with traditional olfactory feedback technologies, the odor generators reported by Liu et al. based on the physical phase change of odorous paraffin wax by controlling the heating temperature exhibit advances in the realization of 32 odor with adjustable concentrations and long operation duration to support long-term utilization without frequent replacement. In addition, the whole system is the first to be built on a skin-integrated soft substrate and equipped with a paired intelligent electronic control panel, allowing remote operation of various selective odor types according to users’ requirements.

By leveraging the advanced olfactory feedback integrated with wearable/flexible technologies^13,14^, the high-channel odor generation arrays of a miniaturized, lightweight, and flexible format expand the interactive potential of VR applications, facilitating experiential learning and emotional modulation (Fig. 2). Users control the odor generation in the virtual world through event-triggering in the real world, which can be better used to help beginners recognize the smell of different types of flowers and plants, and to serve as a teaching function. It can also be used as important feedback in VR games, such as picking different fruits in a virtual orchard and simulating flower arrangements. Moreover, the importance of olfactory generation technology lies in its capacity to enhance immersion and realism by simulating various scents that evoke emotional responses and deepen cognitive engagement. While VR primarily caters to visual and auditory senses, adding olfactory stimuli enriches the sensory experience, fostering a more profound sense of presence within the virtual environment. Olfactory cues have been shown to trigger vivid memories and emotional reactions, thus contributing to heightened cognitive and emotional engagement. Olfactory feedback can also realize olfactory training to ameliorate the disorders resulting from various causes, such as upper respiratory tract infections, trauma, idiopathic factors, and neurological diseases^14^. Furthermore, research suggests that incorporating a greater variety of odor in the training regimen can enhance the rate of improvement in olfactory function. The future of olfactory feedback is advancing towards olfactory encoding^15^, by leveraging neuroscientific analysis to achieve the mapping from chemical structures to olfactory perception and microelectronics technology to realize quantified feedback for complex mixed odor. Developing bidirectional AI algorithms for enhanced odor recognition and odor generation in a system aiming at advanced metaverse applications is the next milestone technology. By combining olfactory cues with visual, auditory, and tactile feedback, virtual environments can be made more comprehensive and lifelike, expanding the application field across various domains, including entertainment, education, and healthcare, offering users a more diverse and immersive sensory experience. For example, in a fire escape practice, the fidelity and effectiveness of training can be achieved by providing the smell of smoke based on the odor generator technology from Liu et al.^13,14^ and high-temperature sensation based on thermal-tactile feedback^3,5^. In the future, the advanced micro/nano-scale technology may help to create new olfactory generators and required electronic control devices for metaverse applications.

**Fig. 2:**
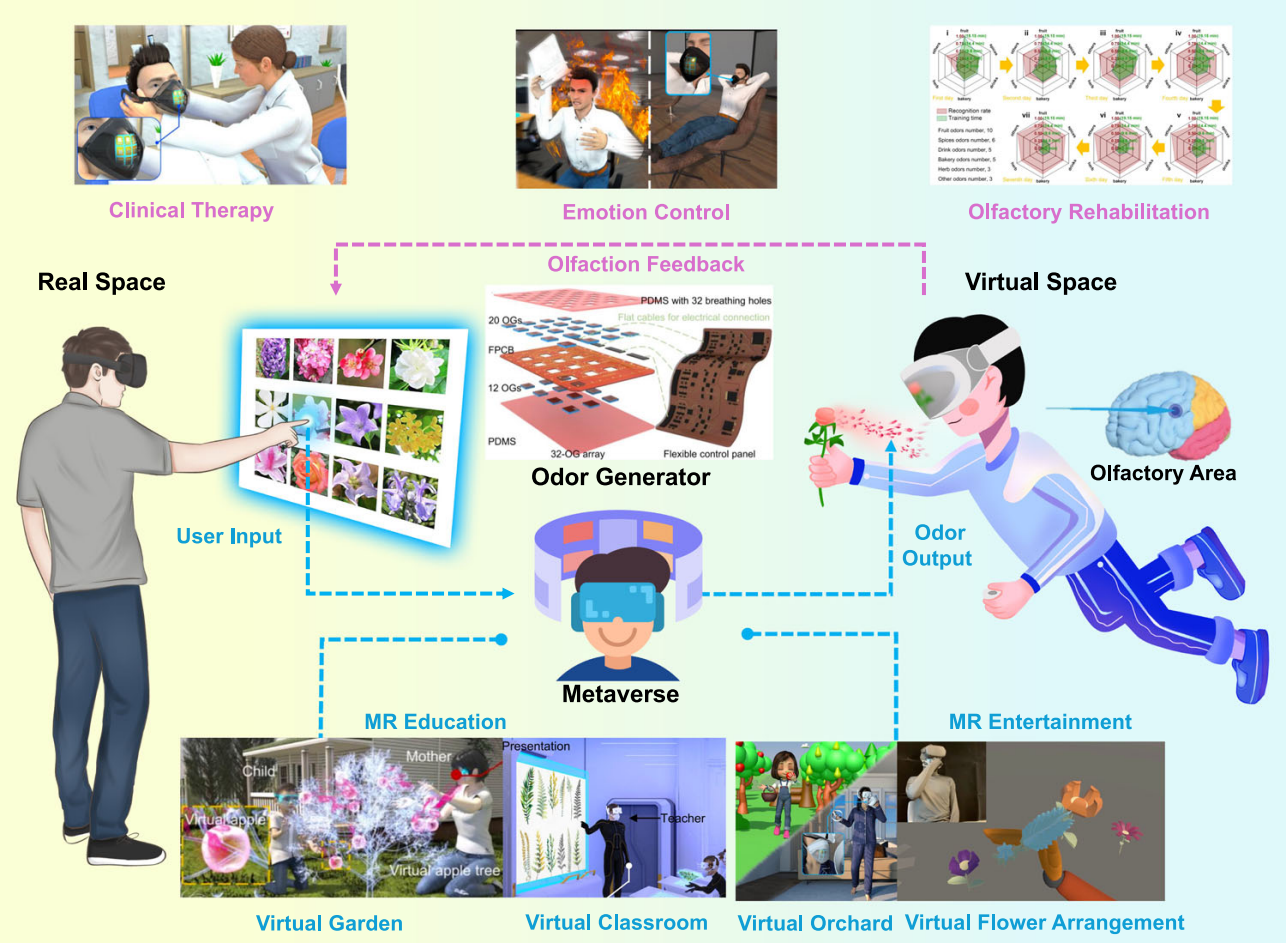
Virtual reality applications of wearable olfactory interfaces. The implementation of different odor releases from odor generators bridges the gap between reality and virtual worlds, applied in mixed reality for education and entertainment^13,14^. Various odor releases in virtual spaces can serve clinical therapy, emotion regulation, and olfactory training for olfactory disorders.

## References

[1] Sun, Z., Zhang, Z. & Lee, C. A skin-like multimodal haptic interface. *Nat. Electron.* **6**, 941–942 (2023). 10.1038/s41928-023-01093-w

[2] Chung, H. U. et al. Skin-interfaced biosensors for advanced wireless physiological monitoring in neonatal and pediatric intensive-care units. *Nat. Med.* **26**, 418–429 (2020). 32161411 10.1038/s41591-020-0792-9 PMC7315772

[3] Lee, J. et al. Stretchable skin‐like cooling/heating device for reconstruction of artificial thermal sensation in virtual reality. *Adv. Funct. Mater.* **30**, 1–11 (2020).

[4] Sun, Z., Zhu, M., Shan, X. & Lee, C. Augmented tactile-perception and haptic-feedback rings as human-machine interfaces aiming for immersive interactions. *Nat. Commun.* **13**, 5224 (2022). 36064838 10.1038/s41467-022-32745-8 PMC9445040

[5] Huang, Y. et al. A skin-integrated multimodal haptic interface for immersive tactile feedback. *Nat. Electron.* **6**, 1020–1031 (2023). 10.1038/s41928-023-01115-7

[6] Iitani, K., Toma, K., Arakawa, T. & Mitsubayashi, K. Transcutaneous blood VOC imaging system (skin-gas cam) with real-time bio-fluorometric device on rounded skin surface. *ACS Sens.* **5**, 338–345 (2020). 31874557 10.1021/acssensors.9b01658

[7] Liu, M. et al. A star-nose-like tactile-olfactory bionic sensing array for robust object recognition in non-visual environments. *Nat. Commun.* **13**, 79 (2022). 35013205 10.1038/s41467-021-27672-z PMC8748716

[8] Li, D. et al. Machine learning-assisted multifunctional environmental sensing based on a piezoelectric cantilever. *ACS Sens.* **7**, 2767–2777 (2022). 36106454 10.1021/acssensors.2c01423

[9] Ren, Z., Zhang, Z., Wei, J., Dong, B. & Lee, C. Wavelength-multiplexed hook nanoantennas for machine learning enabled mid-infrared spectroscopy. *Nat. Commun.* **13**, 3859 (2022). 35790752 10.1038/s41467-022-31520-z PMC9256719

[10] Liu, X. et al. Artificial intelligence-enhanced waveguide “photonic nose”- augmented sensing platform for VOC gases in mid-infrared. *Small* **2400035**, 1–12 (2024). 10.1002/smll.202400035 38576121

[11] Nakamoto, T., Ito, S., Kato, S. & Qi, G. P. Multicomponent olfactory display using solenoid valves and SAW atomizer and its blending-capability evaluation. *IEEE Sens. J.* **18**, 5213–5218 (2018). 10.1109/JSEN.2018.2834953

[12] Yang, P. et al. Self-powered virtual olfactory generation system based on bionic fibrous membrane and electrostatic field accelerated evaporation. *EcoMat* **5**, 1–15 (2023). 10.1002/eom2.12298

[13] Liu, Y. et al. Soft, miniaturized, wireless olfactory interface for virtual reality. *Nat. Commun.* **14**, 2297 (2023). 37160931 10.1038/s41467-023-37678-4 PMC10169775

[14] Liu, Y. et al. Intelligent wearable olfactory interface for latency-free mixed reality and fast olfactory enhancement. *Nat. Commun.* **15**, 4474 (2024). 38796514 10.1038/s41467-024-48884-z PMC11128017

[15] Lee, B. K. et al. A principal odor map unifies diverse tasks in olfactory perception. *Science.* **381**, 999–1006 (2023). 37651511 10.1126/science.ade4401 PMC11898014

